# Translational biomarker discovery in clinical metabolomics: an introductory tutorial

**DOI:** 10.1007/s11306-012-0482-9

**Published:** 2012-12-04

**Authors:** Jianguo Xia, David I. Broadhurst, Michael Wilson, David S. Wishart

**Affiliations:** 1 Department of Biological Sciences, University of Alberta, Edmonton, AB Canada; 2 Department of Medicine, University of Alberta, Edmonton, AB Canada; 3 Department of Computing Science, University of Alberta, Edmonton, AB Canada; 4 National Research Council, National Institute for Nanotechnology (NINT), Edmonton, AB T6G 2E8 Canada

**Keywords:** Biomarker analysis, ROC curve, AUC, Confidence intervals, Optimal threshold, Sample size, Bootstrapping, Cross validation, Biomarker validation and reporting

## Abstract

Metabolomics is increasingly being applied towards the identification of biomarkers for disease diagnosis, prognosis and risk prediction. Unfortunately among the many published metabolomic studies focusing on biomarker discovery, there is very little consistency and relatively little rigor in how researchers select, assess or report their candidate biomarkers. In particular, few studies report any measure of sensitivity, specificity, or provide receiver operator characteristic (ROC) curves with associated confidence intervals. Even fewer studies explicitly describe or release the biomarker model used to generate their ROC curves. This is surprising given that for biomarker studies in most other biomedical fields, ROC curve analysis is generally considered the standard method for performance assessment. Because the ultimate goal of biomarker discovery is the translation of those biomarkers to clinical practice, it is clear that the metabolomics community needs to start “speaking the same language” in terms of biomarker analysis and reporting-especially if it wants to see metabolite markers being routinely used in the clinic. In this tutorial, we will first introduce the concept of ROC curves and describe their use in single biomarker analysis for clinical chemistry. This includes the construction of ROC curves, understanding the meaning of area under ROC curves (AUC) and partial AUC, as well as the calculation of confidence intervals. The second part of the tutorial focuses on biomarker analyses within the context of metabolomics. This section describes different statistical and machine learning strategies that can be used to create *multi*-*metabolite* biomarker models and explains how these models can be assessed using ROC curves. In the third part of the tutorial we discuss common issues and potential pitfalls associated with different analysis methods and provide readers with a list of nine recommendations for biomarker analysis and reporting. To help readers test, visualize and explore the concepts presented in this tutorial, we also introduce a web-based tool called ROCCET (ROC Curve Explorer & Tester, http://www.roccet.ca). ROCCET was originally developed as a teaching aid but it can also serve as a training and testing resource to assist metabolomics researchers build biomarker models and conduct a range of common ROC curve analyses for biomarker studies.

**Electronic supplementary material:**

The online version of this article (doi:10.1007/s11306-012-0482-9) contains supplementary material, which is available to authorized users.

## Introduction

Biomarkers are objectively measurable biological characteristics that can be used to diagnose, monitor or predict the risk of disease (Atkinson et al. 2001). For example, BRCA1 mutations are genetic markers for breast cancer risk (Miki et al. 1994), blood glucose is a standard chemical biomarker for monitoring diabetes, serum creatinine is a chemical marker for kidney function, and prostate specific antigen (PSA) is a protein biomarker for prostate cancer (Polascik et al. 1999). As “omics” technologies such as transcriptomics, proteomics and metabolomics have emerged, the possibility of both measuring and using multiple biomarkers simultaneously to predict or diagnose disease has captured the imagination of many clinicians and scientists. Certainly it is common practice among physicians to use multiple physiological biomarkers (age + BMI + triglyceride level + cholesterol level = cardiac disease risk) to improve the sensitivity and specificity of a clinical diagnosis. Therefore it stands to reason that by combining two or more biomarkers together it might be possible to generate more accurate diagnoses and prognoses or better distinguish between similar diseases (Newby et al. 2001). This, of course, is the motivation behind many recent biomarker studies in metabolomics. Fundamentally, the goal of biomarker development in metabolomics is to create a predictive model from a collection of multiple compounds, which can be used to classify new samples/persons into specific groups (e.g. healthy vs. diseased) with optimal sensitivity and specificity. From a statistics and machine learning point of view, there are three major steps involved in biomarker analysis—(1) biomarker selection, (2) performance evaluation, and (3) model creation. *Biomarker selection* involves the identification of an optimal subset of features that will provide the maximal discriminating power between the diseased and healthy samples. *Performance evaluation* involves the assessment and validation of the panel of biomarkers proposed by step one. *Final model creation* involves developing a fixed mathematical equation or computer algorithm, which combines the panel of selected biomarkers into a *single test score* with the aim of accurately predicting a particular clinical outcome, given measured biomarker responses from a particular target population. Steps one and two are often iteratively combined.

Current metabolomics studies can be placed into two general categories—those that aim to understand biological processes and those that aim to develop biomarkers. Studies in the first group focus primarily on gaining improved biological understanding through the analysis of metabolite profiles. Data analysis is usually performed using multivariate statistical methods such as principal component analysis (PCA) or partial least squares discriminant analysis (PLS-DA) (Trygg et al. 2007). These dimension reduction methods summarize and transform 100s–1,000s of metabolite features into a few key components that capture the maximal variance or discriminatory covariance in the data. A 2D or 3D scatter plot of these components is usually presented to describe the overall patterns of change, or latent structure in the data, under different conditions. The results are accompanied by a relatively long list of compounds that were selected based on a given model’s loading values, variable importance in projection (VIP) scores, or alternatively *p*-values derived from parametric univariate hypothesis testing (Student’s *t* test, ANOVA etc.) or their non-parametric equivalent (Mann–Whitney *U* test, Kruskal–Wallis etc.) performed, in turn, on each measured metabolite. In most cases, these kinds of statistical analyses are not sufficient to acquire detailed biological understanding. As a result, researchers often resort to functional analyses that incorporate prior biological knowledge to help reveal key underlying biological processes. For example, metabolite set enrichment analysis (Xia and Wishart 2010b) or metabolic pathway analysis (Xia and Wishart 2010a; Gao et al. 2010; Kankainen et al. 2011) can be performed on these long compound lists, and the results can be used to infer possible biological processes. While these compound lists are sometimes referred to as “putative biomarkers” by some authors, they are not really useful as clinical biomarkers, which require somewhat different analysis, evaluation and validation procedures. In other words, the analytical methods used by those wanting to understand biological processes differ fundamentally from those wanting to discover or develop biomarkers. These differences are outlined below.

In contrast to metabolomic studies focused on deciphering biological processes, where interesting metabolites are found post hoc, in biomarker studies metabolite selection should be performed a priori rather than post hoc. That is, biomarker selection must be performed before deriving a definitive multivariate predictive model. Furthermore, whereas long lists of metabolites or large multivariate models amalgamating 100s of molecular features are quite useful for understanding pathways and biological processes, they are not ideal for developing cost-effective biomarker tests. Rather, a short list of 1–10 biomarkers is mathematically much more robust and far more practical for clinical testing purposes. While pattern discovery methods such as unsupervised clustering or PCA are useful for discovering novel biological processes, they are not ideal for biomarker discovery. Instead, supervised machine learning algorithms, or multivariate regression models should be used to build the predictive models needed for biomarker analysis. That is, for biomarker discovery one needs to use methodologies that model the discriminatory relationship between a binary dependent variable *y* (typically a two-state clinical outcome variable such as healthy vs. diseased) and one or more explanatory variables *X* (in this context a list of metabolite features). Performing biomarker selection based on univariate statistical significance is equally inappropriate, as often metabolites that are not significant in isolation can, when combined into a single multivariate model, produce clear and reproducible discrimination. Likewise, a significant difference in the average levels of a metabolite between two patient groups does not necessarily mean that the given compound will be a good classifier/biomarker. Perhaps the most important difference to remember is that biomarker models are not intended to help explain biology. Rather they are designed only to discriminate with an optimal sensitivity/specificity without regard to biological cause or biological interpretation. In other words, biological understanding is not an absolute prerequisite for biomarker development. However, understanding the underlying biological pathways certainly can give some rationale to support an assay or give some direction to develop a treatment.

## Overview of biomarker studies in metabolomics

**Fig. 1:**
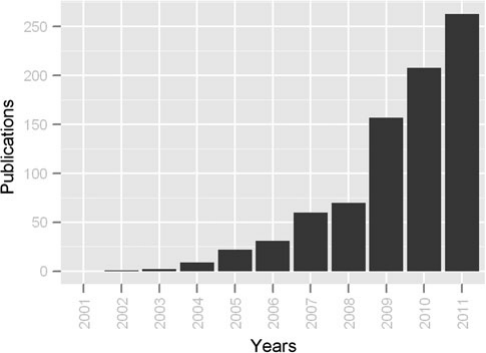
PubMed search results using key words “metabolomics” and “biomarker” from year 2001 to 2011

In some respects, metabolomics has already been remarkably successful in seeing biomarkers translate to the clinic. MS/MS-based screening for inborn errors of metabolism (IEM) in neonates is now routinely done in most industrialized countries (Chace 2001). These high throughput methods measure dozens of metabolites simultaneously (esp. amino acids and acylcarnitines) and are able to diagnose more than 30 different disorders (Wilcken et al. 2003). While most clinical chemists would not confess to performing metabolomics, the principles and technologies behind newborn screening and metabolomics biomarker testing are largely the same. The advantages of using metabolite biomarkers (speed, reproducibility, quantitative accuracy, low cost, non-invasiveness, small sample volume) combined with the remarkable success of newborn screening programs worldwide has inspired many metabolomics researchers to pursue biomarker studies for other diseases. Figure 1 shows the number of annual publications containing both “metabolomics” and “biomarker” in the last 10 years (2001–2011) based on PubMed search results. From 2001 to 2008, there was a slow but steady increase from zero to 70 publications per year. Since 2009, a rapid growth has occurred with over 250 papers published on metabolomics-based biomarker studies in 2011.

While the interest in metabolomic biomarkers has been growing almost exponentially and biomarker discovery efforts have been continuing for >10 years, the number of metabolomics-based tests available for non-IEM diseases stubbornly remains at “zero”. There are three probable reasons for this. First, unlike most chronic or common diseases, IEMs can often be diagnosed using only a single metabolic marker. This is because the concentration differences for that biomarker, between normal and diseased, are so profound that the test sensitivity/specificity is often 100 %. On the other hand, non-IEMs (i.e. common, chronic diseases) exhibit considerably smaller concentration changes spread among of dozens of metabolites, making the development of accurate, single compound tests almost impossible. The second reason has to do with the general lack of quantitation in many metabolomics assays and in most metabolomics biomarker studies. Nearly every approved clinical test, including IEM tests, measures chemical (or protein) concentrations in absolute terms (nM, μM or mM). Unfortunately, compound quantification has not, historically, been a priority in many metabolomics labs. This may be due to the fact that compound quantification is both difficult and time-consuming (although it is now getting much easier). The third reason—which may be the most important—has to do with the general lack of know-how in how many metabolomics researchers perform and report biomarker studies. Based on our review of the literature, there is remarkably little consistency and relatively little rigor in how metabolomics researchers select, assess or report their candidate biomarkers. For instance, many studies identify and report only individual biomarkers in a qualitative way (up vs. down or present vs. absent) without any explicit description of changes in metabolite concentration (or fold-change) together with associated confidence intervals. Biomarker studies that are slightly more quantitative will often report the performance of multivariate models using arbitrary “correct classification” criteria; however no statistical measures of reliability or clinical applicability are provided. PLS-DA models are routinely employed in metabolomics biomarker research where often the coefficient of determination (R^2^) and cross-validated R^2^ (Q^2^) are presented as measures of clinical utility, rather than in their true role (i.e. as a method for choosing the optimal model structure and simultaneously guarding against model over-fitting). R^2^ and Q^2^ performance measures can certainly be used as part of the biomarker selection process; however, they provide very little transparency and they are not a readily interpretable indication of the clinical utility of a given model for a target population. Additionally, most end users (i.e. clinicians or clinical chemists) are not familiar with this style of model evaluation and hence are very skeptical of such an approach. Remarkably few studies present receiver operator characteristic (ROC) curves. Indeed only 15 out of the 823 (<2 %) publications on metabolomics and biomarkers in the last 10 years mentioned the term ROC. This is surprising given that for binary classification problems (e.g. disease vs. healthy), ROC curve analysis is generally considered the standard method for describing and assessing the performance of medical diagnostic tests (Obuchowski et al. 2004; Zweig and Campbell 1993; Pepe et al. 2001). If the ultimate goal is to move metabolite biomarkers from the benchtop to the bedside, metabolomics researchers need to speak the same language as their target audience in order to effectively communicate their findings.

The primary goal of this tutorial is to introduce some basic techniques commonly used in clinical biomarker analysis and to provide some practical guidance on how to apply these concepts to metabolomic data. The advice and recommendations we provide here are primarily intended to apply to human biomarker studies with a special focus on translating these discoveries to the clinic. Common issues, misuses, and pitfalls will also be discussed. We will conclude the tutorial with a brief introduction to an online tool we have recently implemented as a teaching aid that supports and implements some relatively simple, yet practical approaches covered in this tutorial. The intended audience for this tutorial includes bench researchers and clinicians who are interested in biomarker discovery using metabolomics-based technologies. The methods and principles discussed here primarily apply to the discovery and validation of diagnostic, prognostic, predictive and monitoring biomarkers for human disease, for human toxicity and for human studies involving drug monitoring and drug efficacy.

*Note* In this tutorial, for clarity and simplicity, the term ‘biomarker’ or ‘biomarker score’ refers to either a single biochemical measurement (e.g. metabolite concentration) or a predictive score from a multivariate model combining several biochemical measurements (e.g. a multi-metabolite biomarker model). The key point to remember is that in both these situations the data generated for a set of test subjects (biological specimens) will be a single explanatory variable whose values will be real and continuous. In this sense, a multi-metabolite biomarker score can be considered the equivalent to a single metabolite concentration.

## ROC curve analyses in clinical chemistry

Most clinical chemistry tests are applicable to a dichotomous or binary outcome, meaning that they categorize subjects into two states: positive or negative, disease or no disease, admitted or discharged. For a continuous biomarker measurement (e.g. metabolite concentration) the decision as to which outcome a given test subject is categorized is typically based on some pre-determined concentration or detection threshold. In predictive biomarker studies, the performance of a candidate biomarker is determined by comparing the *predicted* outcome to the *true* outcome for a representative set of subjects sampled from a target population. The true outcome is typically determined by monitoring the subjects after the biological specimen has been collected to see if the clinical outcome is ultimately diagnosed to be positive or negative based on some well-established clinical signs or physiological measurements. In general, the nature of this *true classification* process is dependent on the clinical application of the biomarker and a thorough discussion is beyond the scope of this paper. However, what ultimately results is a dependent “outcome” variable (a positive or negative class label) to which a biomarker “score” can be compared.

The performance of a given biomarker can be assessed in several ways. The simplest, and most naïve, method is to quote the percentage correctly classified. This is known as the *predictive accuracy*. This approach is flawed in several fundamental ways. Firstly, it forces the developer of the biomarker to predetermine the optimal decision boundary (critical biomarker concentration/score) from which subjects will be classified as having either a positive or negative outcome. It may well be that the mathematically optimal threshold is not the optimal clinically useful threshold. For example, it may be an ethical necessity for *all* positive outcome subjects to be correctly classified at the cost of many subjects being incorrectly negatively classified. The choice of the optimal decision threshold should be determined jointly with domain experts such as physicians and health economists before being transferred to the end user (i.e. the testing labs). Secondly, biomarker discovery studies are often performed on a small (*n* < 100) but representative sample drawn from a given target population. This means that there will always be some uncertainly in the predictive accuracy of any reported test (recall the smaller the sample, the larger the uncertainty). Thus, presenting a single measure of accuracy without any associated statistical measure of uncertainty is simply bad scientific practice. Indeed, it is comparable to reporting a sample mean without an associated standard error. Confidence intervals and sample size will be discussed, in detail, later. Finally, it is important to note that predictive accuracy is not a reliable metric for the real performance of a biomarker if the sample population is unbalanced (that is, when the number of subjects in different classes varies greatly). This is generally the case in clinical settings, where (hopefully) most people are healthy and very few are diseased. For example, if the prevalence of a positive outcome in a given population is low, say five in every 100 subjects, and a biomarker is presented that *always* predicts a negative outcome, then this biomarker would be considered 95 % accurate, which is misleading. The problem of outcome imbalance can be avoided by retrospectively designing a matched nested case–control study from an existing larger prospective study (typically using bio-banked specimens). A matched nested case control study is a variation of a case–control study in which only a subset of controls from the larger cohort are compared to the disease cases. This type of experimental design and the associated issues are discussed in detail elsewhere (Dunn et al. 2011, 2012; Rothman and Greenland 1998). Using this approach, even in extremely unbalanced target populations, a balanced biomarker discovery study can be designed and conducted. Regardless of how one designs and conducts a biomarker “discovery” project, ultimately any candidate biomarker test must be validated in a large cross-sectional study so understanding the limitations of different performance metrics remains very important.

A far superior approach to the assessment of biomarker performance is to consider the frequency with which the test produces: true positives (TP), true negatives (TN), false positives (FP) and false negatives (FN). One then summarizes these values into the proportion of actual positives that are correctly classified as positive (sensitivity) and the proportion of actual negatives that are correctly classified as negative (specificity). In the context of a biomarker designed to discriminate between diseased and healthy subjects:

TP: the number of diseased subjects that are correctly identified as disease (outcome positive & test positive)
TN: the number of healthy subjects that are correctly identified as healthy (outcome negative & test negative)
FP: the number of healthy subjects that are incorrectly identified as diseased (outcome negative & test positive)
FN: the number of diseased subjects that are incorrectly identified as healthy (outcome positive & test negative)

**Fig. 2:**
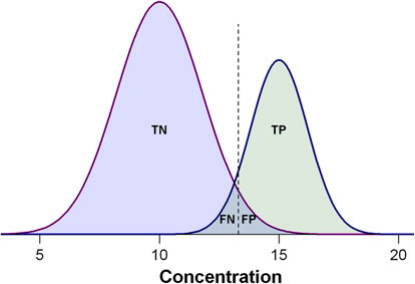
Illustration of TP, TN, FP, and FN with hypothetical biomarker test data. The distributions of true outcomes are given by the two Gaussian curves with positive cases on the right side and negative cases on the left. The cut-off level is indicated by the dashed line. Due to the overlap between the biomarker concentrations of the two populations, the cut-off level will misclassify the left-hand side of the positive cases and the right-hand side of the negative cases. *TP* true positives, *TN* true negatives, *FP* false positives, *FN* false negatives

The definitions of TP, TN, FP and FN are illustrated in Fig. 2. And sensitivity (Sn) and specificity (Sp) are mathematically defined as:

- Sn = TP/(TP + FN)
- Sp = TN/(TN + FP)

For ease of interpretation, sensitivity can be considered as the probability of a positive test result given that a subject has an actual positive outcome, and specificity can be considered as the probability of a negative test result given that a subject has an actual negative outcome. Thus, for a given biomarker with a fixed decision boundary (metabolite concentration or model score) a sensitivity of 0.95 and a specificity of 0.6 indicate that: given a new test subject with unknown clinical outcome, when the resulting test score is above the decision boundary there is a 95 % chance that the subject is correctly classified as a positive outcome; but if the test score is below the decision boundary then there is only a 60 % chance that the subject is correctly classified as a negative outcome. It is important to note that this is only true if the new test subject is drawn from the same target population as that sampled to develop the biomarker (i.e. biomarker performance is population specific). Biomarkers designed for a specific population (e.g. pregnant women) are only applicable to that target population.

The sensitivity and specificity of a test can vary depending on the biomarker decision boundary one chooses to classify subjects as either “positive” or “negative”. Changing the decision boundary may, for example, increase the sensitivity at the expense of lowering the specificity, or vice versa. One of the best ways to observe how a decision threshold affects sensitivity and specificity is through a ROC curve. A ROC curve shows how the sensitivity and specificity change as the classification decision boundary is varied across the range of available biomarker scores. Unlike prediction accuracy, a ROC curve is not dependent on the prevalence of a given outcome. Furthermore, because it depicts the performance of a biomarker test over the complete range of possible decision boundaries, it allows the optimal specificity and associated sensitivity to be determined post hoc. Unlike the popular R^2^ and Q^2^ metrics, a ROC curve is a non-parametric measure of biomarker utility rather than a parametric measure of deviation from an ideal model. As a result, when one evaluates a biomarker using a ROC curve there is no need to be worried about the “data-normality” of either the predicted positive or negative score distributions, nor whether the two distributions have equal number of subjects and equal variance. These considerations are very important when using a parametric performance metric. As a result, ROC curve analysis is widely considered to be the most objective and statistically valid method for biomarker performance evaluation (Obuchowski et al. 2004; Zweig and Campbell 1993; Pepe et al. 2001; Soreide 2009).

### Generation of ROC curves

In this section, we provide a simple example on how to generate a ROC curve from the results of a *single* biomarker diagnostic test as might commonly be found in clinical chemistry. However, it is important to note that the process is identical for interpreting the predictions from a fixed multivariate model (i.e. a multi-biomarker test). Here, we use data from a hypothetical 2-h oral glucose tolerance test (OGTT) adapted from Lasko et al. (2005) where the glucose concentration is the continuous or graded value (Table 1).

**Table 1:** Calculation of sensitivity and 1-specificity for each cut-off

Glucose	Diagnosis	Sensitivity	1-Specificity
		1.00	1.00
4.86	Healthy	1.00	1.00
5.69	Healthy	1.00	0.90
6.01	Healthy	1.00	0.80
6.06	Healthy	1.00	0.70
6.27	Healthy	1.00	0.60
6.37	Healthy	1.00	0.50
6.55	Healthy	1.00	0.40
7.29	Healthy	1.00	0.30
7.29	Diseased	0.90	0.30
7.82	Healthy	0.90	0.20
9.22	Diseased	0.80	0.10
9.79	Diseased	0.70	0.10
11.28	Diseased	0.60	0.10
11.83	Diseased	0.60	0.10
12.06	Healthy	0.50	0.00
18.48	Diseased	0.40	0.00
18.5	Diseased	0.30	0.00
20.49	Diseased	0.20	0.00
22.66	Diseased	0.10	0.00
26.01	Diseased	0.00	0.00

Glucose concentrations (mmol/L) are sorted from low to high. Here we assume values above the threshold will be positive (diseased) and below the threshold are negative (healthy)

**Fig. 3:**
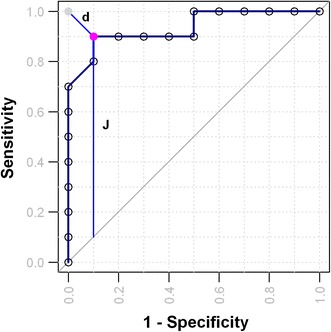
Empirical ROC curve and optimal cut-off. After obtaining a list of sensitivity and specificity values from all possible cut-offs, one should plot all pairs of sensitivity and 1-specificity values as *empty circles*, and then connect each neighboring circles with line segments to generate empirical ROC curves. The optimal cut-off (*solid circle* in *magenta*) can be identified as the point with by minimal *d* the distance from a cut-off to the solid grey circle (0, 1), or the point with maximal vertical distance from the diagonal line, also known as the Youden index *J* (Color figure online)

To generate a ROC curve, we first sort the glucose concentration values in ascending order. Each concentration value in this list essentially represents a different cut-off point. Note, an empty row is inserted at the top of the table to indicate a threshold that is lower than the smallest value. We now calculate the sensitivity and specificity (actually 1 − Sp) for each concentration value (or cut-off) assuming values that are equal or above the current threshold are predicted positive (diseased) and values below the threshold are predicted negative (healthy). After obtaining these values (Table 1, last two columns), we generate a scatter plot with circles representing each pair of sensitivity and 1 − specificity values. We can then obtain the empirical ROC curve by connecting each circle with straight-line segments. The result is shown in Fig. 3. It displays the sensitivity of a diagnostic test over all possible false positive rates (1–Sp). From this example it is clear to see that ROC curves are very straightforward to generate and widely applicable to any two-class distribution of data. Note the jagged shape of the curve due to the small number of data points. ROC curves can be smoothed by adding more measurements or by applying approximation methods using either a kernel density or binormal distribution (Zou et al. 1997; Zweig and Campbell 1993).

### Area under the curve (AUC), ‘optimal’ threshold point and partial AUC

ROC curves are often summarized into a single metric known as the: Area under the curve (AUC). AUC can be interpreted as the probability that a diagnostic test or a classifier will rank a randomly chosen positive instance higher than a randomly chosen negative one. If all positive samples are ranked before negative ones (i.e. a perfect classifier), the AUC is 1.0. An AUC of 0.5 is equivalent to randomly classifying subjects as either positive or negative (i.e. the classifier is of no practical utility). It can be shown that the area under the ROC curve is closely related to the Mann–Whitney *U* test [the nonparametric equivalent of the Student’s *t* test (Bamber 1975)]. The AUC of an empirical ROC curve can be easily calculated using the trapezoidal rule. A rough guide for assessing the utility of a biomarker based on its AUC is as follows: 0.9–1.0 = excellent; 0.8–0.9 = good; 0.7–0.8 = fair; 0.6–0.7 = poor; 0.5–0.6 = fail.

ROC curves are often used to determine the ‘optimal’ cut-off point based on which subjects will be classified as either a positive or negative outcome. There are three common approaches to calculate the optimal points. The first criterion is to minimize the distance to top-left corner (0, 1). As the distance (*d*) from the top-left corner to any point on the ROC curve can be expressed as: *d* = sqrt [(1 − Sn)^2^ + (1 − Sp)^2^] we can calculate the value of *d* for each cut-off point and then locate the point that has smallest value. The second approach is to identify the point with furthest vertical distance from the diagonal line. The point, also known as Youden index: J = max {Sn − Sp − 1} (Youden 1950), can be easily identified by searching for the point with maximal sum of sensitivity and specificity values from all plausible sum values for each cut-off. The first two approaches are illustrated in Fig. 3. As we have discussed earlier, the mathematically optimal threshold may not be appropriate in all clinical applications due to ethical, economic and prevalence constraints. In general, these considerations can be formulated into a single cost function and each cut-off can then be evaluated to identify the point that minimizes the cost. For more discussions and example formulas, please refer to the paper by Zweig and Campbell (1993).

**Fig. 4:**
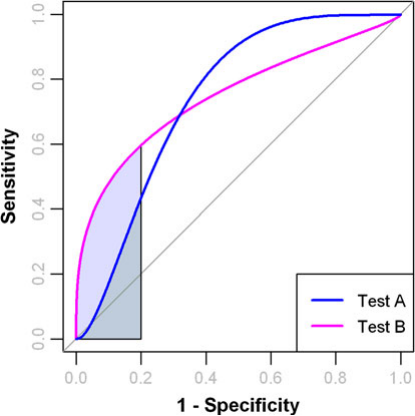
Performance comparison using partial AUC. The AUC of *Test A* and *Test B* are about the same. However, *Test B* is superior to *Test A* at regions of high specificity (0.8, 1). Therefore, using the partial AUC will be more appropriate in this case

AUC is widely used for performance comparison across different biomarker models. However, using the whole area under a ROC curve may not be appropriate in some cases. An example is shown in Fig. 4 in which two diagnostic tests give nearly the same AUC value. However, Test A performs better than test B in regions of high sensitivity, while test B performs better when high specificity is required. This can have very different implications regarding which test should be used or which biomarker should be chosen. Using the partial AUC (pAUC) is most useful when only certain regions of the ROC space (i.e. high sensitivity or high specificity) are of particular interest (Walter 2005; Dodd and Pepe 2003; McClish 1989).

### Confidence intervals

Sensitivity, specificity, ROC curve shape, optimal cut-offs, AUC and pAUC are all estimations of biomarker performance based on limited data sets or limited data sampling. Typically biomarker discovery studies are relatively small (*n* < 100) when compared to the size of the proposed target population (potentially millions of subjects). As such, any performance measure is a sample approximation to the (unmeasurable) performance of the biomarker applied to the target population as a whole. Just as one should always quote a standard error when calculating sample means, with all the metrics described in this tutorial one should always provide confidence intervals (CIs). Typically 95 % CIs are calculated for ROC analysis. It is important to note that a reported 95 % CI *does not* predict that the *true* population statistic has a 95 % probability of falling within the calculated interval. Rather, it describes the range of values the sample statistic will take, with a probability of 0.95, if the identical experiment is repeated many times using independent subjects drawn from the identical target population. For example a reported AUC of 0.8 with 95 % CI of ±0.1 actually means that if one repeated the experiment 100 times, for 95 of those experiments the AUC would lie between 0.7 and 0.9. That said, 95 % CIs provide a very good range of estimates for the unknown true statistic and it is correct to say that “we calculated with 95 % confidence that the true AUC of biomarker X is with the range 0.7–0.9”.

**Fig. 5:**
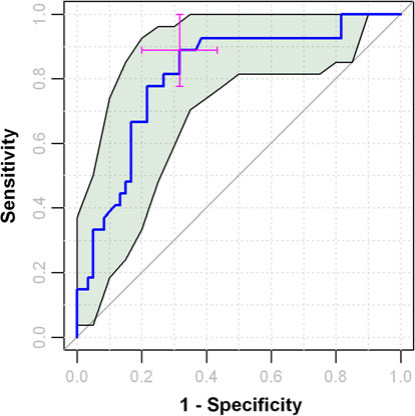
Using a bootstrapping approach to compute the 95 % confidence interval (CI) for a single cut-off or for the complete ROC curve

Many different approaches have been proposed to calculate CIs for ROC curves (Lasko et al. 2005). One straightforward and widely applicable method that we recommend is a technique called bootstrap percentile re-sampling (Carpenter and Bithell 2000; Efron 1987). Bootstrap resampling is a very simple but powerful method of estimating confidence intervals for any population statistic without either having to repeat the experiment in question over and over, or being dependent on parametric estimation of associated standard errors. This is be achieved by simply constructing a number of different samples (called “re-samples”) from the observed dataset, each of which is obtained by random sampling with replacement from the original dataset, such that each sample is equal in size to the observed dataset. The sample statistic is then calculated for each of the re-samples (e.g. total number of re-samples = 1,000), and the 95 % CIs are calculated by simply taking the 2.5 and 97.5 percentiles of the ranked list of the 1,000 values. In this way confidence intervals for the AUC can be readily calculated. Additionally, by utilizing a nearest neighbor approach (or smoothing multiple ROC curves), for any given fixed specificity, a 95 % CI can be calculated for the associated sensitivity, or vice versa. Indeed, given the available computational power today, 95 % CI curves can easily be constructed for the complete ROC curve itself as shown in Fig. 5. Most modern software tools (including ROCCET—see sect. 7) have at least some re-sampling methods implemented that allow users to calculate confidence intervals for certain key parameters.

### Sample size

The sample size used in a particular biomarker discovery study is intrinsically linked to the confidence interval of the generated ROC curve. As with any population-based statistic, the uncertainly associated with a specific ROC curve decreases as the number of individuals tested increases. Similarly, the uncertainty in the ROC curve decreases the more effective the biomarker is (i.e. the higher the AUC the lower the uncertainty). With an AUC of 1, the calculated confidence error will be very close to zero. Consequently, as uncertainty is partially dependent on expected biomarker performance, the choice of sample size is subjective. It can either depend on what the end-user considers a clinically useful result, with a specified minimum requirement, or if the researcher is competing with an existing “gold standard” biomarker, the biomarker under evaluation should, at a minimum, show equal performance. Sample size calculation based on ROC curves has been discussed in several publications (Eng 2003, 2004; Obuchowski et al. 2004). Often, however, the prerequisite is for a test to have a fixed specificity with a minimum sensitivity. In this case a minimum sample size can be estimated using very simple inferential approach introduced by Arkin and Wachtel (1990). For a study in which we hypothesise that a clinically effective case/control screening test will be seen to have a fixed specificity of 0.95 and is expected to have at least a sensitivity of 0.85 and assuming a 95 % confidence interval in sensitivity of ±0.05 is sufficiently precise, it can be calculated that we will require at least 196 cases. If the minimum expected sensitivity is increased to 0.95 the minimum sample size decreases to 73 cases. If the minimum expected sensitivity decreases to 0.7 then the minimum sample size increases to 323 cases. Finally, if the minimum sensitivity is unchanged (0.85) but the required 95 % confidence interval is relaxed to ±0.1, the minimum sample size decreases to 49 cases. As seen by these examples, the minimum sample size can vary a great deal depending on the required utility of the resulting biomarker (See Appendix A for the mathematical formulas as well as example analyses). Note: these sample size calculations are applicable to both single measurement biomarkers and multivariate models. However, these are the *minimum* requirements. Care must be taken in the design of the experiment such that the target population for which the biomarker is aimed are suitably represented in both the positive and negative outcome groups. In extremely heterogeneous target populations, positive outcome individuals must be selected with great care and matched one-to-one (as closely as possible) with negative outcome individuals. Often with extremely rare or low-prevalence diseases it is advisable to over-sample from the negative outcome target population in order to obtain some degree of diversity in the test sample (e.g. match each disease case to four random healthy controls). Also if cross-validation (introduced later in this tutorial) is to be performed then multiplying the minimum sample size by approximately 1.5 is good practice in order to compensate for sub-sampling—although this increase in sample size is purely an empirical recommendation suggested by the authors based on past experience and not on any theoretical justification.

## ROC curve analysis of metabolomics biomarkers

### Multiple comparisons

Unlike the situation for classical clinical chemistry, metabolomics typically involves measuring hundreds of metabolites at a time rather than just one or two. So in principle, metabolomics allows the researcher to evaluate multiple metabolite biomarkers against a given outcome in a single experiment. In this sense a researcher treats metabolomics as the equivalent of performing hundreds of individual clinical chemistry tests, simultaneously. Thus it is possible, using the protocol described earlier, to calculate ROC curves for each compound, and then select potential biomarkers based on those exhibiting the highest AUC or pAUC (allowing for the uncertainty described by the associated confidence intervals). This approach is perfectly valid. However, care must be taken when performing multiple evaluations in a single experiment. The probability of finding a random association between a given metabolite and the outcome increases with the total number of comparisons. In another words, the more metabolites measured in a single experiment the more likely a random association will be found. In this case, a biomarker or set of biomarkers is discovered which is of some clinical utility based on the sample population in question, but that utility disappears when the experiment is repeated multiple times using independent samples drawn from the target population. In this regard, the confidence intervals calculated using the resampling simulation method are not accurate (neither are the parametric equivalent methods). These “false discoveries” are known as *false positives*. A simple method for compensating for multiple comparisons (called Bonferroni correction) involves increasing the percentage confidence levels as a function of the number of comparisons. For example, if the experiment compares 50 metabolites in a single experiment, the acceptable confidence level is changed from 95 to 99.9 % (100(1–0.05/50)). Thus a biomarker that has an AUC 95 % CI of 0.6–0.8, and is therefore is potentially clinically useful, could have a Bonferroni corrected AUC CI of 0.5–0.85 which drastically increases the uncertainty that the biomarker will have any real clinical utility. Bonferroni correction is considered a *very* conservative method for compensating for multiple comparisons, and has the potential for easily throwing-away real biomarkers (*false negatives*) as the number of metabolites measured increases to many thousands. There are several alternative methods to multiple comparison correction such as the Benjamini–Hochberg false discovery rate or FDR (Benjamini and Hochberg 1995), but a full discussion is beyond the scope of this tutorial. Instead, the reader is directed to the following excellent reviews on false discovery and false positives in metabolomics (Broadhurst and Kell 2006; Noble 2009). It is important to stress that by far the best way to avoid false positives is to repeat the experiment (of similar sample size) on independent samples drawn from the same target population. This is known as a validation experiment. If a biomarker displays potential clinical utility in both the “discovery” and “validation” experiments then the researcher can be reasonably confident that this biomarker is worth developing and testing in a much larger clinical trial—or that it can even be moved into clinical practice. The more validation experiments performed the more confidence is accrued.

### Multivariate biomarker discovery

Although it is completely valid to treat a metabolomics experiment as an opportunity to test many hundreds of potential individual (univariate) biomarker compounds in a single experiment, often what results is a long list of quite “weak” biomarkers (AUC < 0.7) with fairly wide confidence intervals. This may be sufficient to imply some sort of epidemiologically significant association between biological mechanism and adverse outcome; however the results may not be strong enough to use any of these individual biomarkers as clinically useful biomarker test.

In multifactorial diseases (such as heart disease, cancer, or neurological disorders) it is often the combination of multiple “weak” individual markers into single a “strong” multivariate model that provides the required high levels of discrimination and confidence. In classical clinical chemistry this is not as easy as it sounds. Trying to perform multiple experiments on the same sample population is time consuming and can introduce many measurement errors. Furthermore, exhaustively adding or multiplying multiple compound concentrations together in various combinations, and then testing the resulting “score” with ROC curve analysis, is a very inefficient method for searching for an effective multifactorial predictive model. Likewise, the probability of finding false discoveries increases dramatically when compared to testing individual markers.

Metabolomic studies, combined with modern multivariate data analysis methods, allow us to perform this multifactorial biomarker discovery in a highly efficient manner. Because many hundreds of compounds are measured in parallel (as a single “snapshot” of metabolism) a metabolomics experiment provides an individual *metabolic profile*, or *fingerprint*, for each analysed individual. A population of these profiles can be converted into an *n* by *m* data matrix (*n* individuals, *m* metabolites), which can then be analysed by computer using methods known as *data projection* or *machine learning*. Here the computer algorithm looks for correlated structure in the measured data that also correlates with the target outcome. The result is a multivariate mathematical equation, or computer program, which provides a single score (derived from multiple biomarkers) analogous to those discussed throughout this tutorial. This score can be assessed through ROC curve analysis as previously described. In addition to the biomarker score, these computational methods generally produce a measure of the importance for each metabolite in the resulting algorithm, which in turn gives an indication of the contribution that each metabolite adds to the model’s performance. In general, the higher the absolute score, the more influential the metabolite. In regression-based methods (e.g. PLS, linear or logistic regression) the importance of a given metabolite can be directly interpreted from the model’s *loadings* vector. The biomarker discovery process is now transformed into discovering a suitably parsimonious subset of these “important” metabolites (biomarker signature) that, in combination with the projection algorithm, produces a ROC curve of sufficient utility.

There are many potentially useful data projection and machine learning methodologies available for this task. Some of the most popular methods that have been applied to metabolomic studies are: linear discriminant analysis (LDA), PLS-DA, decision trees (e.g. CART), random forests (RF), artificial neural networks (ANN), and support vector machines (SVM) (Cortes and Vapnik 1995; Barker and Rayens 2003; Breiman 2001; Eriksson et al. 2001; Trygg et al. 2007).

Unfortunately the use of computationally intensive modelling algorithms can easily be abused. This can lead to the very real possibility of discovering multivariate projections that randomly correlate highly with the test outcome - thus giving a false impression of the true predictive ability of the candidate biomarker signature. This is known as model over-fitting, or “fitting a model to noise”. Careful cross-validation procedures are imperative to avoid this problem. This will be discussed in detail later.

There are four steps in the multi-metabolite or multivariate metabolomics biomarker discovery process: (1) data pre-processing, (2) biomarker selection, (3) performance evaluation, and (4) final model creation. We will describe these steps in a little more detail below and then present some easy-to-use on-line tools to help readers explore and perform these steps in the final section of this tutorial.

### Data pre-processing

#### Sample-to-sample normalization

Often in metabolomics experiments there is unwanted sample-to-sample variation. For example, in urinary metabolomics studies there can be significant dilution effects due to individual’s fluid intake prior to sample collection. In these cases we suggest that some sort of normalization technique be used to equalize this effect (often called *row* or *sample* *normalization*). There are many available techniques, the simplest being to normalize to a single metabolite level (e.g. Creatinine for urine samples). Alternatives, such as probabilistic quotient normalization (Dieterle et al. 2006) and quantile normalization (Bolstad et al. 2003) are proving to be more generally applicable to a broader range of metabolomics data. A comprehensive comparison of state-of-the-art sample-to-sample normalization techniques has recently been published (Kohl et al. 2012).

#### Data filtering

Most metabolomics platforms can simultaneously measure hundreds or even thousands of metabolites in a single experiment. However, only a small proportion of these metabolites will typically exhibit changes correlated with the conditions under study. The observed variations for the majority of the measured metabolites simply reflect random fluctuations around a baseline response. Before embarking on the selection of multiple biomarkers, it is important to initially filter out clearly non-informative metabolites that will never contribute to the final biomarker panel. This step is very important for high-dimensional metabolomics data, but often underappreciated by many researchers. Prudent data filtering has the potential of reducing the computational cost as well as improving the power to identify real biomarkers (Hackstadt and Hess 2009; Bourgon et al. 2010). Non-informative metabolites can be characterized into three groups: (1) those exhibiting very small values close to the limit of detection; (2) those in which the given metabolite are only detected in very few specimens; and (3) those that are near-constant irrespective of the difference in clinical outcome. The identification of those metabolites belonging to the first category requires some platform-specific knowledge, whereby concentrations below a specified limit of detection are set to “missing”. Again missing values are used to represent features that are not detected in a given specimen. Metabolites with more than a user-defined percentage of missing values (typically 20 %) should then be removed from the data set, and the remaining values replaced with some small values (i.e. their lower detection limits) or estimates derived from a missing value imputation algorithm. Finally, the low variance features can be detected using the standard measure of relative variance known as the relative standard deviation, RSD (the sample standard deviation divided by the sample mean). An RSD < 15 % is usually sufficiently invariant to warrant removal. However, depending on the reproducibility of the analytical platform of choice, researchers may want to choose a higher/lower threshold. Specific threshold values need to be determined empirically but the measurement scientist.

#### Data transforming

All parametric statistical methods assume that that the data has come from a specific type of probability distribution, and then make inference based on the parameters of that chosen distribution. If the data under examination does not hold to that distribution, then the inferences can be false, or at best misleading. Methods popular in the metabolomics community (ANOVA, MANOVA, CVA, LDA, PLS-DA) assume that the data comes from a Gaussian distribution. Or to be more precise they assume that a given model’s *residuals* are normally distributed with a homogeneous variance. Residuals are estimates of experimental error obtained by subtracting the observed outcome from the estimated outcome (positive and negative outcome often being represented as the numerical values +1 and −1 respectively). Therefore, in order for any statistically meaningful model to be produced from metabolomics data it advisable to transform the data before modelling. Although there is no fixed protocol, the standard practice for metabolomics data is to perform logarithmic transformation (i.e. replace each value, *x*, with log_10_(*x*)). This has the effect of monotonically reducing extremely high values, which in turn produces homoscedastic and near-normal or near-Gaussian model residuals. Other monotonic transforms have also proved useful (e.g. power transforms such as square root, or cube root) and a more detailed discussion can be found in a recent paper on the subject (van den Berg et al. 2006). Note that non-parametric data analysis methods, such as those based on decision trees (e.g. CART and RF) do not require data transformation.

#### Data scaling

For a given biofluid specimen (e.g. human serum) the average abundance of the many metabolites found therein can vary by several orders of magnitude. As a result, highly abundant compound species can dominate a projection model and obscure small but potentially important biomarkers during the downstream multivariate analysis. Therefore, data scaling is another very important step in biomarker discovery. Data scaling methods divide each data point for a given metabolite by a scaling factor that is usually some measure of data dispersion for that feature. In most cases, scaling is also applied together with data centering. The most popular scaling method is “autoscaling” (also known as standardization or unit variance scaling) in which the data for each metabolite is mean centred (subtract the sample mean from each data point) and then divided by the sample standard deviation. The result is that the data for each metabolite will have a unit mean and unit standard deviation, and thus each metabolite can be compared with no bias due to absolute abundance. It is important to note that autoscaling is a very sensitive to large deviations from the sample mean as outlying samples can totally skew the scaling coefficients. It is therefore often sensible to perform outlier detection and data transformation before data scaling. There are several popular alternative scaling methods (such as Pareto scaling or Range scaling) and again a more detailed discussion can be found in the paper by van den Berg et al. (2006).

### Biomarker selection

As discussed earlier, in order to implement a cost-effective and reproducible clinical test, a multivariate projection model utilizing 100s of molecular features is not ideal. Mathematically this may be feasible, but developing a single assay to reproducibly quantifying many hundreds metabolites for use in a hospital clinical chemistry laboratory is an extremely difficult task and often impractical. Developing an assay based on a short list of 1–10 biomarkers is a far more attractive proposal and any subsequent computational algorithm is likely to be mathematically much more robust.

For metabolomics biomarker discovery this means that, within the modelling process, it is important to find the simplest combination of metabolites that can produce a suitably effective predictive outcome. This is not a simple task, as the biomarker discovery (feature selection) process now involves optimizing two parameters: (1) the biomarker utility—AUC etc. and (2) the number of metabolites used in the predictive model. Multi-objective optimization problems such as these have been the subject of intensive studies in the bioinformatics and machine learning communities for many years (Handl et al. 2007; Knowles et al. 2001). Here we provide a very high-level overview of the two most popular approaches applicable to metabolomics. Also, the final section of this tutorial presents some easy-to-use on-line tools to explore and perform biomarker selection. For a more comprehensive review and discussion, please refer to two excellent review papers (Isabelle and Andr 2003; Saeys et al. 2007).

#### Feature selection using filters

This is the simplest and most widely used method of feature selection in metabolomics studies. As described earlier most projection models provide both an overall model prediction score and a variable importance score. Feature filtering simply involves ranking the variables used in the model in order of importance, and then repeating the modelling process using the top *N* metabolites. Each subset model is then evaluated, producing the requisite ROC curve. The investigator then subjectively chooses the optimal value for *N* such that an adequate ROC curve is produced. Although this method is very straightforward it does require the investigator to have a good understanding of the underlying modelling algorithm, and may require some manual editing of the variable importance list before ranking. There is no theoretical guarantee that the top *N* variables from the full model will produce the optimal subset model (of the same complexity). This is particularly true for projection methods such as PLS-DA, where the process of projecting the metabolite responses into a latent structure of reduced dimensionality means that there is an inherent information compression, which although optimizing the model performance at the complete fingerprint level, does not necessarily optimize the model at the parsimonious metabolite level. That said, this very quick and methodologically transparent method of variable selection can often produce a model with the prerequisite performance.

#### Feature selection using wrappers

Another popular approach to biomarker selection is known as the *wrapper* method. Methods falling under this category are wholly data-driven and require no direct interpretation of a model’s parameters (or variable importance score) and are independent of the chosen modelling methodology. The simplest of these methods is *Forward Selection*. In this approach, starting with no variables, one adds variables to the model, one at a time. At each step, each variable that is not already in the model is tested for inclusion in the model. The variable that improves the model’s prediction most significantly is added to the model. The variable addition process is repeated until a set maximum number of variables is reached or when there is no significant improvement to the model. A similar approach called *Backward Elimination* starts with the full model and then removes variables showing the smallest contribution to the model, one at a time. A modification of the forward selection technique, known as *Stepwise Selection*, combines both of the above approaches. As with forward selection, variables are added to the model one at a time; however after a variable is added, the stepwise method looks at all the variables currently included in the model and deletes any variable which is no longer significantly contributing to the model’s performance. Alternately, a more computationally intense selection method known as *subset selection* can be used. This method does not settle for a single “best” model, but instead tries to find the best one-variable model, the best two-variable model, the best 3-variable model, etc. up until the best *N*-variable model. Subset selection is performed either by exhaustively searching all combinations of available variables or, as this becomes mathematically intractable when the number of available variables is large, optimal subsets are searched using a heuristic methodology such as *genetic algorithms* (Broadhurst et al. 1997; Jarvis and Goodacre 2005).

It is important to note that depending on the statistical or machine learning method one uses for filtering or feature selection, it may be necessary to optimize each individual candidate model’s structure (e.g. number of latent variables in a PLS-DA model) to avoid over-fitting. This is done using cross-validation and is discussed in the next section. However, the final result will be one or more fixed model(s) (i.e. fixed variables subset, fixed model structure, and fixed parameter values) which will also have to be independently validated outside the feature selection process as a whole.

### Performance evaluation

#### Cross-validation

It is imperative that the performance of any biomarker selection process be independently evaluated so that over-fitting is avoided. The easiest approach for cross-validation (CV) is to create what is known as a holdout set. The available data set is split into two parts, the *training set* and the *hold*-*out set* (or *test set*). Typically the hold-out set is selected to be 1/3 of the total data, and is randomly stratified such that it suitably represents the training set (i.e. equal proportion of outcomes, and similar demographics etc.). In this way ROC curve analysis of a biomarker model based on both the training set and hold-out set can be performed. The true performance of the biomarker model can *only* be judged by the holdout set ROC curve analysis.

Often biomarker discovery studies unavoidably involve small sample numbers (e.g. less than 100 individuals) and/or the sample populations are heterogeneous making it difficult to effectively split the resulting data into two suitably representative sets. In these situations methodologies have been developed in order to evaluate how a given model’s predictive ability will generalise to an independent data set without actually creating a holdout set. These methodologies are known as CV. A single round of CV involves partitioning a sample of data into two subsets, performing the model optimization on one subset (training set), and evaluating the model performance on the other subset (validation set). To produce a realistic estimate of model performance, multiple rounds of CV are performed using different partitions, and the performance results are averaged over the rounds. Common types of CV include repeated random subsampling (e.g. Monte Carlo sampling), k-fold, and leave-one-out CV (Picard and Cook 1984; Eriksson et al. 2001; Efron and Tibshirani 1997).

#### Nested cross-validation

Generally the biomarker selection process involves two levels of model validation; one to optimize the model structure given for each candidate subset of variables, and the other to validate the variable selection process as a whole. Again if the investigator is dealing with small sample sizes then *nested CV* can be performed. The simplest way to explain this process is to use Monte Carlo cross-validation as an example. Firstly, step-1, the complete data set is randomly split into training and test sets as described above. Then, step-2, the filter/wrapper biomarker selection is iteratively performed using *only* the training set, whereby each candidate variable-subset model is evaluated using CV (this is the nested CV). The result will be an optimal parsimonious model, which is then independently validated using the test set. The performance of this optimal model is judged solely on the ROC curve analysis of the *test data*. Step-1 and step-2 are then repeated *N* times such that *N* optimal model evaluations are performed. By inspection of the feature subsets selected across all *N* optimal models (typically with a histogram) the investigator can determine whether a consistent panel of metabolite biomarkers has been found. By inspection of the *N* model parameter-sets the investigator can determine whether a consistent model structure has been determined. By inspection of the *N* different ROC curves, the investigator can determine the value and consistency of the predicted outcome. A full technical description of nested cross validation, also known as double CV, and its various subtle variations is beyond the scope of this tutorial but these issues are discussed in detail elsewhere (Westerhuis et al. 2008; Filzmoser et al. 2009; Liebmann et al. 2010; Smit et al. 2007; Szymanska et al. 2012).

#### Permutation testing

A second level of model validation can be performed using a technique known as permutation testing (Good 2011). In permutation testing, the null hypothesis to be proved or disproved is that the optimal model found during the biomarker discovery process could also have been found if each patient sample had been randomly assigned a clinical outcome (positive or negative) in the same proportion as the true assignment. In this test, the model structure and variable subset is fixed, and multiple “randomly permuted” models evaluated (e.g. *N* = 1,000). This results in a reference distribution of the null hypothesis. The “true” (correctly assigned) model performance is then statistically compared to this reference distribution and a *p* value calculated. A *p* value <0.05 means that given a randomly permuted outcome variable there is less than a 5 % chance that a model of similar performance to the “true” non-permuted model will be produced.

Cross-validation and permutation approaches offer different measures of a biomarker model’s utility. Permutation testing indicates whether a given model is significantly different from a null model (random guessing) for the sample population while CV gives an indication of how well a given model might work in predicting new samples. In other words, permutation testing validates the proposed model structure; while CV validates the generalizability of the model. For example, a biomarker model can give significant *p* value in permutation tests but perform poorly in CV tests. On the other hand, a model with reasonable performance based on CV could fail permutation tests (Westerhuis et al. 2008). These two measures are complementary to each other and both should be performed when evaluating a multi-component biomarker model (Bijlsma et al. 2006; Xia and Wishart 2011).

### ROC curve analysis for biomarker discovery

**Fig. 6:**
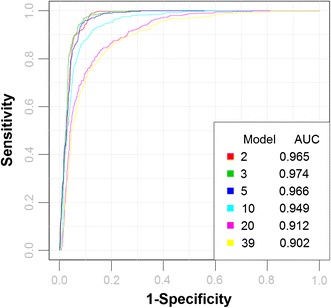
Comparison of different models based on ROC curves. Six biomarker models were created using a linear SVM with different numbers of features. ROC curves were generated using the predicted class probabilities from repeated cross validation for each model. The legend shows the feature numbers and the AUCs of the six models

ROC curve analysis for model performance during biomarker discovery differs from the ROC curve analysis of a fixed biomarker score (as described earlier in this tutorial) in one fundamental way. During CV not only are the optimal subset of metabolites being selected, but also the optimal parameter values for the associated modelling procedure are estimated. In particular, each iteration of the CV process produces different model parameter values, and hence potentially a different range of model prediction values. So when ROC curve analysis is performed on the multiple CV test sets, for a given candidate biomarker model, a family of ROC curves are produced, which can then be averaged to produce a smooth curve. Figure 6 shows a set of ROC curves for SVM models created using different subsets of metabolites selected using the filter approach. As with the fixed biomarker ROC curve analysis, confidence intervals can also be generated for the cross-validated models using a variation of the bootstrap methodology described earlier. In this instance the averaged CV predicated ROC curve is used as the test metric, rather than the ROC curve generated by using *all* the data on a fixed model. In this way the confidence interval reflects both the uncertainty in the sampling procedure and also the uncertainty in the parameter optimization.

### Model creation

Once the biomarker discovery phase is complete, and hopefully a suitably robust and effective set of metabolites has been defined, then the last stage of the process is to generate the final fixed biomarker model (or computer algorithm). This is done using all the available data for the metabolite subset, applied to the optimal model structure. Essentially it is a process of finalizing, or fixing, the optimal model parameters. It is possible that the modeling method used to “discover” the metabolite biomarkers is not the best method to “translate” the biomarkers into clinical practice. For example the optimal subset of biomarkers may be determined by PLS-DA, but more effectively translated (i.e. improved performance) using a simpler model such as logistic regression. Additionally, a researcher may find it preferable to change the analytical platform used to measure the chosen final metabolite list. For example the discovery phase experiment may have been performed using an untargeted LC–MS protocol; whereas once the metabolites have been definitively identified it may be preferable to measure the metabolite responses using a more sensitive targeted instrument such as a triple quadrupole LC/MS/MS system. Changes in both technology and model selection are perfectly acceptable in practice, as long as a suitable validation experiment is performed. This validation process is discussed in the next section.

Once the model parameters are fixed, a final ROC curve analysis can be performed to define its performance. It is at this point that one may wish to define the optimal decision boundary for classifying samples as either being positive or negative outcome. This should be done under close consultation with physicians, disease-specific experts, health economists and other end users. This decision boundary will then be used to define the final model’s specificity and sensitivity (with confidence intervals).

#### Parameter confidence intervals

Great care must be taken to make sure that the final biomarker model is mathematically robust, particularly if the sample size is small. In other words, the final model parameter values must be very stable. This assumption can be tested using bootstrap resampling of the complete data set. Here, for each resample the model is optimized and its parameter values recorded. Then, based on multiple resamples (e.g. *N* = 1,000), 95 % confidence intervals for each of the parameter values are estimated. Evaluation of these confidence intervals can be somewhat subjective. However, if the standardized variance is high (>20 %) and the confidence range close to zero, then one may want to reassess the biomarker discovery process as a whole before moving forward. After all the required statistical analysis has been performed, and all confidence intervals have been determined to be within a given tolerance, then the final model needs to be validated experimentally. A detailed discussion of this process is beyond the scope of this tutorial, however the various kinds of experiment validation are worth introducing.

*Note* For publication it is imperative that the results of the nested CV be reported, as this provides the most realistic indication of the biomarker utility. The final model creation step is used to simply fix the final model parameters and to assess whether the model is robust enough for further repeat experiment validation. Once repeat experiments have been conducted then the performance of the final model on this new data must be reported and used as the realistic measure of clinical utility.

### Repeat and replicate biomarker validation

Validation experiments can be performed in several ways, or more exactly, with several levels of imposed experimental variability. The simplest is a lab repeatability study, where replicate specimens are used. In this kind of study the metabolite measurements are performed on the identical instrument, by the same observer, in the same lab. The degree of agreement of the ROC curves can then be compared between the two repeat studies. If quantified metabolite concentrations are measured then the degree of agreement in biomarker concentration for a given test subject can be compared.

The second level of validation is a lab replication study. Here independent samples are drawn from the same target population. The number of samples should be no fewer, than the “discovery” experiment. Then these samples should be analysed on the same instrument as the discovery study and, if possible, using the same observer. Comparisons at this stage can be used to remove false positives.

The third level of validation is an inter-lab repeatability study that again uses replicate specimens from the individuals that were measured in the discovery experiment, but the validation experiment is performed in a different lab using a different instrument (potentially the same manufacturer) and a different observer. Comparisons to the previous studies can determine any increased variability due to independent lab practices.

The fourth level of validation is an inter-lab replication study. Again, a new set of test subjects are drawn from the target population; however, this time, as with level three, the experiment is performed in an independent lab. If the comparison of ROC curves, and measures of reproduction of individual metabolite concentrations, are within a specified tolerance and remain at a level that indicates clinical benefit then the selected biomarkers can be considered strong enough to withstand scrutiny of a formal clinical trial. If the inter-lab variability is outside the tolerance limits then it may be worth investigating an alternative, potentially more stable, analytical platform or assay for the discovered biomarkers.

## Comments and common pitfalls

### Potential issues in data filtering a feature selection

Due to the high-dimensional nature of most ‘omics’ data, including that coming from metabolomics experiments, data filtering procedures are often used before a biomarker model is created. In the data pre-processing section, we described some standard methodologies based on using the intensities or relative variances of features. In these methods, no outcome information is used in the filtering process (non-specific filtering). However, improper use of data filtering proved to be a very problematic issue during the early days of microarray data analysis (Ambroise and McLachlan 2002). This issue has also been frequently seen in metabolomics. A typical example is that a *t* test is first used to filter out non-significant features and then the remaining features are used to build a predictive model. This approach can easily produce very good results. It is then claimed the model can be used to for disease diagnosis with very high accuracy. This assertion is not justified because the model was essentially evaluated on the same dataset that was used to select biomarkers. In other words, information about the class labels was already “leaked” during the filtering step without any sort of validation to avoid false discoveries. To correct for this bias, a predictive model must be evaluated using different datasets that have not been used at either the feature selection stage or the model training stage. We suggest that one first perform non-specific filtering as described earlier, and then perform an embedded feature selection procedure inside each iteration of CV.

### Implications of normalization procedures

Data scaling (and centering) procedures involve the use of population parameters estimated from the data (i.e. mean or standard deviation) to improve data conformity to yield better performance. This approach works very well in CV, when all the data (training or testing in each split) are more likely drawn from the same distribution. However, this is not necessarily the case in the real-world applications in which the entire population is usually more diverse than the samples. Therefore, centering and scaling can have a rather negative impact on overall model performance. This issue does not exist for transformation (i.e. log transform) procedures, which can be applied directly on the new values.

### Issues with AUC-based performance evaluation

**Fig. 7:**
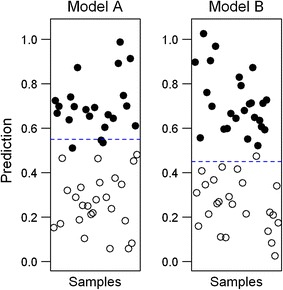
Difference between ranking and classification. The two scatter plots show the predicted class probabilities for 50 new samples by two biomarker models. Both models are able to rank all new samples correctly. Therefore, they both will have the same AUC (1.0) but exhibit different error rates (3/50 and 1/50 respectively) due to their different decision boundaries, which were determined during the model creation process

There are a few common pitfalls associated with ROC based metrics (Obuchowski et al. 2004; Berrar and Flach 2010). In particular, using AUC as the only performance measure for a biomarker model can sometimes be misleading. As discussed earlier, the AUC essentially quantifies the ranking ability of a biomarker model. If all positive samples are ranked before the negative samples, the AUC is 1.0. This only suggests that the model can *potentially* give a perfect prediction on these new samples. In practice, when predicting the outcome for new samples, the decision boundary calculated from the training data may still not be optimal for the new samples. This will lead to errors, even though the rankings are correct. Similarly, two models with different error rates can sometimes give the same AUC. This situation is illustrated in Fig. 7.

When comparing the performance of different models using AUC, a common mistake is to check if the CIs of the AUC overlap. However, the difference between two AUC may be statistically significant even when their CIs overlap. The correct procedure, especially when comparing two groups, is always to calculate the CI for the difference between the underlying ROC curves-i.e. an estimate of the difference in AUC. If the lower bound of the resulting CI is less than zero then there is no significant difference.

### Improper performance evaluation

It is often the case that the performance of a biomarker model reported in the literature cannot be validated in independent studies. Although population heterogeneity can be an important factor leading to poor reproducibility, it is more often the case that improper procedures used in performance evaluation in the original study led to an overoptimistic or misleading assessment of the test’s performance. For instance, CV is often used to determine the best subset of features that give the maximal discrimination for a given modelling algorithm. One common pitfall is that both the feature selection and model evaluation are performed using the same data set; thus the model has been optimized and tested on the same data, which usually leads to unrealistically good performance measures. The correct approach is to have the performance of the model evaluated on independent data. In practice, as described in this tutorial, performing nested CV can approximate external validation. When doing this, one needs to be aware that this result is not actually the performance measure of the final global, fully optimized model. Rather, it reflects the average performance of multiple local optimal models created in each fold based on the training data. These models can be very different especially if the sample size is small or very heterogeneous. As the sample size increases, the local optimal models created in each fold will gradually converge, and the performance will be close to the actual performance of the final optimal model.

## A list of recommendations for biomarker reporting

Throughout most of this tutorial we have provided advice, commentary and explanations regarding the selection and analysis of both single and multiple biomarkers for disease diagnosis and/or prediction. However, we also think it is important to provide some recommendations on how to implement these ideas, especially with regard to reporting, publishing and implementing biomarkers or biomarker models. Here is a list of nine recommendations that summarize some of the key points of this tutorial.

1. Record and report absolute concentration data where possible. Remember that nearly all approved clinical tests require absolute concentration data.
2. Biomarkers must consist of positively identified compounds. Unknowns or tentatively identified features cannot (and never will) be approved for clinical laboratory testing.
3. Report details on the sample size (i.e. the size of testing, training and validation samples), population characteristics and features of the diseased and healthy populations along with any relevant metabolomics data.
4. Report the classification or biomarker modelling method(s) used, the validation steps performed and confidence intervals of the final biomarker model.
5. Minimally, report the sensitivity/specificity of the biomarker(s) or biomarker model. Ideally ROC curves with confidence intervals should be provided and plotted.
6. If possible provide the equation(s), rules, algorithm parameters or software code (as supplementary material) used to generate the biomarker model.
7. Compare the performance of your biomarker(s) or biomarker model to previously existing methods using appropriate quantitative or statistical methods.
8. Recent court decisions suggest that biomarkers derived from naturally existing genes, proteins or metabolites are not very patentable. In other words, no research group is likely to get rich from discovering a biomarker—but they may become famous. The best way of getting a useful biomarker or set of biomarkers into practice is to collaborate with clinical chemists or clinical microbiologists and to work with them to rigorously validate and verify the biomarker(s) on “approved” (Clinical Laboratory Improvement Amendments, CLIA or other regulatory bodies) equipment. Once suitably validated, biomarkers can be used in the clinic. Hiding key information about biomarkers or biomarker models will inevitably slow down their translation to the clinic and limit their benefits to the intended population.
9. Given that suitable ethical consent is granted, and given any intellectual property rights have been secured (i.e. upon publication of results), we strongly encourage researchers to deposit their raw quantitative (or semi-quantitative) metabolite data onto an online repository such as Metabolights (www.ebi.ac.uk/metabolights/) (Sansone et al. 2012) so that other researchers can verify, or improve upon, the presented research.

## ROCCET: an online tool for ROC based biomarker analysis

Given the complexities and challenges of biomarker analyses, it is highly recommended that trained bioinformaticians or biostatisticians familiar with the field should carry out biomarker analysis. However, many clinicians and bench researchers who are interested in conducting biomarker analysis do not have ready access to these resources. Likewise, there are many who would like to learn how to perform these kinds of analyses themselves or at least have enough knowledge to ask the right questions or go to the right people. This tutorial was developed to help these individuals. In particular, we have tried to give a simplified overview of the common methods, suggestions and pitfalls associated with clinical biomarker analysis, especially with regard to multi-biomarker analysis. Some of the techniques may be simple to grasp, but hard to implement—especially if one is not a computer programmer or statistician. Other concepts may need to be seen, explored or tested interactively to really come to a full understanding of their strengths or limitations. To this end, we have developed a relatively simple and user-friendly online tool—ROCCET (ROC Curve Explorer & Tester, http://www.roccet.ca) to help readers better understand the methods and ideas described in this tutorial. ROCCET is intended to serve as a teaching/training tool as well as to assist researchers and clinicians who are new to biomarker analysis and who are interested in performing some basic exploratory biomarker selection and modelling.

### Data input and processing

ROCCET has a number of pre-collected, pre-processed and pre-tested data sets (MS and NMR) derived from our own metabolomics research. These data sets are designed to help users test the concepts or visualize the methods described in this tutorial. Users are free to upload their own data as well. ROCCET accepts a compound concentration table (or aligned peak intensity table) with the sample values/concentrations in rows and the feature labels in columns. The second column must always be a set of class (healthy or disease) labels. The data table should be uploaded as a text file in comma separated value (.csv) format that can be easily generated from any spreadsheet program. In order to improve its utility and efficiency in handling different omics data types, a number of utilities have been implemented to perform basic data processing. These include a variety of functions for filtering non-informative features, functions for missing value imputation, as well as various data transformation and scaling procedures. Detailed descriptions of these functions are available on the data processing page and ROCCET’s FAQs page. ROCCET supports (1) classical ROC curve analysis, (2) multivariate or multi-marker ROC curve exploration and (3) ROC curve testing. These modules are described in more detail below:

### Classical ROC curve analyses

This module allows users/readers to explore, visualize and perform the ROC analytical methods described in Sect. 3 of this tutorial including classical ROC curve analyses for single biomarkers or features, as well as: (1) calculation of AUC and CI, (2) identification of optimal thresholds, (3) calculation of sensitivity, specificity, as well as the CIs for any given threshold. The user can interactively adjust many output parameters and display options.

### Multivariate ROC curve explorer

This module aims to help users/readers identify multiple biomarkers and assess their classification performance as outlined in Sect. 4 of this tutorial. ROCCET offers three well-established machine learning or statistical algorithms with built-in feature importance measures: (1) linear support vector machine (SVM), (2) PLS-DA and (3) RF. To estimate the predictive performance as well as the stability of the selected features, a balanced Monte-Carlo cross-validation (MCCV) procedure with 50 iterations is used. In each MCCV, two-thirds of the samples are randomly selected to evaluate the feature importance and the most important features are selected with different cut-offs to build models which are validated on the remaining 1/3 of the samples. These models are assessed by AUC or prediction accuracies. The results are presented in various graphical “views” such as the ROC view, the Prob(ability) view, the Sig(nificant) Feature view, etc. to facilitate their understanding.

### ROC curve tester

This module allows users/readers to manually select one or more features and to create a “custom” classifier using one of the three algorithms described above. The performance of the model can then be evaluated using MCCV procedure. The significance of the model can be further validated using permutation tests. Users can also manually specify “hold-out” sample subsets to test the performance of the model created using the CV procedures.

### Example analysis

Here we present the analysis on a subset of the data from a recently published metabolomics study on early preeclampsia (Bahado-Singh et al. 2012). The data contains 42 metabolite concentrations measured on 90 human plasma samples collected from 30 patients and 60 healthy controls. The purpose of the study was to identify a small number of metabolites that could be used to reliably predict the eventual onset of the disease. This data set is available as one of the test data sets on ROCCET’s web site.

#### Data upload and processing

First, go to the ROCCET home page (http://www.roccet.ca). On the “Data Upload” page, select the first test data set and click “Submit”. The next page shows the summary of ROCCET’s data integrity check. Click “Next” to enter the “Data Processing” page. Use the default selections and click “Submit”. The next page shows a graphical summary of data normalization process. Click “Next” to enter the “Data Analysis” page.

#### Data analysis

The “Data Analysis” page allows users to choose among the three analysis modes which we will explore below. Select the univariate ROC curve analysis and click “Submit”. The page shows all features ranked by their AUC. Click on any compound name, the corresponding ROC curve will be generated. In addition, a box plot overlaid with the optimal cut-off is also displayed. Users can click the “Next” button to view the detailed sensitivity, specificity and confidence intervals for the current selected feature. Using the left navigation tree to return to the “Data Analysis” page, select multivariate ROC curve explorer and then click “Submit” to start ROCCET’s automatic feature selection and performance assessment. The result will return in a few seconds. Various views are displayed through multiple tabs. For example, the default “ROC view” shows the ROC curves of all models under investigation. The “Sig(nificant) Features” view shows important features associated with these models. The default classification algorithm is linear SVM. Users can also choose PLS-DA or RF. Return to the “Data Analysis” page, select the ROC curve tester option and click “Submit”. In the next page, select the top five metabolites then click “Submit”. The performance of linear SVM (the default) is AUC ~0.98 with 95 % CI [0.971–1.00]. Permutation tests show that the model is very significant (*p* < 0.002 based on 500 permutations).

## Summary and conclusions

Most metabolomics researchers are quite familiar with using various multivariate statistical approaches to analyze and interpret their metabolomics data. Biomarker analysis, however, requires a different approach. Based on our review of the literature, our assessment of numerous presentations at many conferences and our discussions with many metabolomics scientists, it appears that when it comes to biomarkers, many researchers are using suboptimal methods with improper performance measures and incomplete reporting standards. To help remedy this situation we decided to write this tutorial and to prepare the ROCCET web-server.

In sect. 1 we discussed some of the key differences and shared similarities between functional metabolomics and biomarker discovery. In sect. 2 we described some of the successes and challenges with regard to biomarker discovery and biomarker implementation in metabolomics. In sect. 3 we introduced ROC curves and discussed their utility in evaluating single biomarker (classical clinical chemistry) tests. In sect. 4 we summarized the advantages and described the methods used for generating and assessing multi-biomarker models. In sect. 5 we discussed the pitfalls and potential shortcomings of some of these biomarker analysis methods. We also provided some examples of common errors seen in many biomarker studies. In sect. 6 we tried to summarize some of these points into a set of eight recommendations regarding the measurement, reporting and implementation of metabolomics biomarkers. Finally in sect. 7 we introduced ROCCET, a web-based tool to help readers visualize, interact and better understand the concepts introduced in sects. 1–5.

Although this tutorial is written with metabolomics and metabolite concentration data in mind, most of the approaches are equally applicable to non-targeted metabolomics data (using relative concentrations instead of absolute concentrations) and to other omics (transcriptomics and proteomics) data as well. This tutorial is not meant to be exhaustive, covering all aspects of biomarker selection or disease risk assessment. Rather, it was designed to focus on the most common approaches and the most practical solutions. We are hopeful that this document and the accompanying software resources will further inspire and educate the metabolomics community. We also hope that this information may help lead to the discovery, validation and clinical implementation of newer or better disease biomarkers that may eventually have a long-lasting impact on human health and quality of life.

## Electronic supplementary material

Below is the link to the electronic supplementary material.

Supplementary material 1 (DOCX 137 kb)
