# Supplementary material for: Translational biomarker discovery in clinical metabolomics: an introductory tutorial
Source: Metabolomics. 2012 Dec 4;9(2):280–99. doi: 10.1007/s11306-012-0482-9 (PMC3608878; doi:10.1007/s11306-012-0482-9)
Supplement: Supplementary file 1 — Supplementary material 1 (DOCX 137 kb) [file 11306_2012_482_MOESM1_ESM.docx]

**Appendix A**

| Performance Metric of Interest | Formula | Symbol Description |
| --- | --- | --- |
| Sensitivity | $\begin{matrix} n_{case}=\frac{Z_{\alpha}^{2}S_{n}(1-S_{n})}{\theta^{2}} \\ n_{total}=n_{case}/P \end{matrix}$ | *S_n_* : Sensitivity;  *S_p_* : Specificity;  *n_case_* & *n_total_* : estimated number of required cases & total subjects  *θ_n_* & *θ_p_* required absolute precision in sensitivity or specificity;  *Z_α_*: standard normal value and $\alpha$ is the confidence level (Z_0.05_ = 1.96 for 95% confidence level);  *P* : prevalence of the disease in population;  *Var(AUC)* : anticipated variance of AUC;  $k:$*k*: ratio of prevalence of case to control subjects |
| Specificity | $\begin{matrix} n_{case}=\frac{Z_{\alpha}^{2}S_{p}(1-S_{p})}{\theta^{2}} \\ n_{total}=n_{case}/(1-P) \end{matrix}$ |  |
| AUC | $\begin{matrix} n_{case}=\frac{Z_{\alpha}^{2}\times Var(AUC)}{\theta^{2}} \\ n_{total}=n_{case}(1+k) \end{matrix}$ |  |

**Commonly used formulas for sample size calculation**

Note:

1. The prevalence of the disease can be obtained from the literature or a pilot study;
2. The AUC variance can be obtained from the literature or estimated using a non-parametric bootstrapping method;
3. If one is interested in more than one performance measure, calculate the sample size for each parameter and then choose the largest sample size;
4. Formulas to calculate sample size for comparing sensitivity, specificity or AUC with a predefined value (i.e. gold standard) are more complex and can be found in the paper by (Obuchowski et al. 2004a).

**An example analysis as described in the accompanying paper:**

“For a study in which we hypothesise that a clinically effective case/control screening test will be observed to have a fixed specificity of 0.95 and is expected to have at least a sensitivity of 0.85 and assuming a 95% confidence interval in sensitivity of +/- 0.05 is sufficiently precise, it can be calculated that we will require at least 196 cases.”

In this case, the performance metric of interest is sensitivity, therefore, the number of cases (subjects with disease) will be:

$$n_{case}=\frac{1.96^{2}\times0.85(1-0.85)}{0.05^{2}}$$

The total number of subjects varies depending on the prevalence of the disease.
